# Characterization of Microbiological Quality of Whole and Gutted Baltic Herring

**DOI:** 10.3390/foods11040492

**Published:** 2022-02-09

**Authors:** Jaana Huotari, Irina Tsitko, Kaisu Honkapää, Hanna-Leena Alakomi

**Affiliations:** VTT Technical Research Centre of Finland, Ltd., P.O. Box 1000, FI-02044 Espoo, Finland; jaana.huotari@vtt.fi (J.H.); irina.tsitko@vtt.fi (I.T.); kaisu.honkapaa@vtt.fi (K.H.)

**Keywords:** 16S rRNA gene amplicon sequencing, MALDI-TOF MS, microbiological quality, whole fish, gutted fish, microbiota

## Abstract

There is growing interest in Baltic herring (*Clupea harengus membras*) and other undervalued, small-sized fish species for human consumption. Gutting or filleting of small-sized fish is impractical; hence, the aim of this study was to explore the suitability of the whole (ungutted) herring for food use. The microbiological quality of commercially fished whole and gutted herring was analysed with culture-dependent methods combined with identification of bacterial isolates with MALDI-TOF Mass Spectrometry and culture-independent 16S rRNA gene amplicon sequencing. Whole and gutted herring had between 2.8 and 5.3 log_10_ CFU g^−1^ aerobic mesophilic and psychrotrophic bacteria and between 2.2 and 5.6 log_10_ CFU g^−1^ H₂S-producing bacteria. Enterobacteria counts remained low in all the analysed herring batches. The herring microbiota largely comprised the phyla Proteobacteria, Firmicutes, and Actinobacteria (71.7% to 95.0%). *Shewanella*, *Pseudomonas*, and *Aeromonas* were the most frequently isolated genera among the viable population; however, with the culture-independent approach, *Shewanella* followed by *Psychrobacter* were the most abundant genera. In some samples, a high relative abundance of the phylum Epsilonbacteraeota, represented by the genus *Arcobacter*, was detected. This study reports the bacterial diversity present in Baltic herring and shows that the microbiological quality was acceptable in all the analysed fish batches.

## 1. Introduction

Demand for fish and fish-based products is rising globally, while many of the undervalued fish species remain underutilized in food production. The annual catch of Baltic herring (*Clupea harengus membras*) in Finland is approximately 100 million kilograms, making herring the most important fish species in the Finnish commercial marine fish catch by volume [1]. Currently, however, the majority of herring is exploited in the domestic market as animal feed with low economic value [2]. Herring is considered a nutritious and sustainable alternative to other protein foods for human consumption [3] and thus offers an attractive raw material for value-added food products [4]. However, due to the small size of Baltic herring, gutting or filleting is not feasible for most of the catch. Processing the whole fish into the final product would therefore enable the use of the small, undervalued fish as food [4]. Thus, the microbiological quality of the whole small-sized herring requires investigation.

In fish, microbes are mostly located in the gills, skin, and intestinal tract but rapidly colonize the flesh after death [5]. The bacterial composition of fresh fish consists of a wide variety of different bacteria, yet primarily those of the psychrotrophic Gram-negative genera such as *Photobacterium*, *Shewanella*, *Psychrobacter*, and *Pseudomonas* [5,6,7]. Storage conditions, e.g., atmosphere and temperature, favour specific spoilage microbes that ultimately dominate the product [8,9]. *Shewanella* spp. and *Pseudomonas* spp. can grow fast during storage on ice in the presence of oxygen and frequently are the major bacterial genera in fish stored in such conditions [7,10,11].

In food microbiology, methods based on cultivation are widely applied to examine the overall microbial quality and to detect the presence of certain microorganisms. The emergence of Matrix-Assisted Laser Desorption Ionization/Time-of-Flight Mass Spectrometry (MALDI-TOF MS) has revolutionized the identification of cultivable microbiota, making feasible the comparison of microbial abundances and relations in cultivable culturable populations [12]. As culture-based methods reveal only a fraction of microbial diversity, culture-independent techniques have emerged as an alternative for the study of the microbiota of foodstuff [13]. Recent studies have taken an approach of combining MALDI-TOF MS with culture-independent 16S rRNA gene amplicon sequencing in assessing the contamination level of pork and poultry meat [14,15,16]. Likewise, the development of next-generation sequencing techniques has provided additional information on fish microbiota and shelf life [7,13].

Several studies have explored the microbial levels of Baltic herring [4,17,18,19,20]; however, little is known about the microbial populations present in this fish species. Research has focused on the large-sized, particularly aquacultured, fish species (e.g., salmon, tilapia, and sea bream [7,13,21]), and hence the microbiota of those commercially valuable fish are well known. The microbiota of the small-sized fish, notably in the Baltic Sea area, have been scarcely documented.

The aim of the study was to investigate the microbiological quality of the small-sized whole (ungutted) herring from the point of view of its suitability for food use as such, in comparison with gutted herring. For that purpose, we analysed several commercially fished herring batches intended for use in the food industry. We used conventional culture-dependent methods combined with MALDI-TOF MS identification of the bacterial isolates and assessed the overall bacterial population with culture-independent 16S rRNA gene amplicon sequencing.

## 2. Materials and Methods

### 2.1. Herring Samples

Whole and gutted (intestines, head, and tail removed) Baltic herring (*Clupea harengus membras*) samples were provided by three Finnish fish wholesalers between March 2019 to November 2020. The warm summer months (July and August) are not represented in this study because there is no commercial catch of herring at that time. The herring samples were caught from the Baltic Sea by trawling a maximum of four days before arriving in the laboratory and were kept on ice at 0 °C before microbiological analyses. The batch obtained on 6 March was kept at 4 °C for 16 h before cultivation. The whole herring averaged 16.3 ± 1.1 cm in size and the gutted herring 10.3 ± 2.1 cm in size (post-gutting). From each batch of herring, three to five fish were examined in parallel. Furthermore, whole and gutted fish were frozen at −80 °C for molecular biology assays.

### 2.2. Culture-Based Microbiological Analyses

Three to five fish in parallel were examined from each fish batch. The fish were analysed as whole and gutted and were homogenised with scissors before sampling. Ten-gram samples were weighed, diluted with 90 mL of peptone saline, and homogenised with a Stomacher (Stomacher 400 Circulator, Seward, Worthing, UK) for 60 s at 260 rpm. A ten-fold dilution series was prepared and plated with the spread plate technique. Table 1 presents the growth media and cultivation conditions.

Enterobacteriaceae counts were estimated based on the percentage of oxidase-negative colonies following the Nordic Food Analysis Committee (NMKL) method 144 [22]. Coliform counts were estimated based on the percentage of colonies confirmed as coliforms by Matrix-Assisted Laser Desorption Ionization Mass Spectrometry (MALDI-TOF MS; Bruker, Bremen, Germany). The colonies from MRS plates were tested for catalase production, and the LAB counts were assessed based on the percentage of catalase-negative colonies.

### 2.3. Identification of Bacterial Isolates

#### 2.3.1. MALDI-TOF MS Analysis

The most abundant colony types (a total of approximately 25 colonies per parallel sample) were picked from PCA, Lyngby iron agar, and Chromocult agar plates with <300 colonies. Pure cultures were prepared from the dominant bacterial colonies and processed for identification with MALDI-TOF MS Biotyper (Bruker Daltonics, Bremen, Germany). Samples were prepared using the direct transfer method according to the manufacturer instructions. The data were processed by the Bruker MBT Compass 4.1 and Flex Control 3.4 software (Bruker Daltonics, Bremen, Germany), and the obtained mass spectra were compared with those in the commercial libraries provided by Bruker (research use only, RUO, database; includes 7311 mass spectra). Score values of ≥2.3 were used for highly probable species-level identifications as described by Jeong et al. [23]. Score values of 1.7 to 2.29 were sufficient for identification at the genus level. Isolates identified with a score value of <1.7 were re-analysed with the extended direct transfer method.

#### 2.3.2. 16S rRNA Gene Sequencing of Bacterial Isolates

Bacterial isolates identified by MALDI-TOF MS with a score of <1.70 were identified with 16S rRNA sequence analysis. Genomic DNA was extracted from pure cultures with the NucleoSpin^®^ Microbial DNA kit (Macherey-Nagel, Düren, Germany). Extracted DNA was amplified with universal primers BSF 8/20 (5′-AGAGTTTGATCCTGGCTCAG-3′) and BSR 1541/20 (5′-AAGGAGGTGATCCAGCCGCA-3′) with My Taq™ HS Red DNA Polymerase (Bioline, London, UK). PCR was conducted in Mastercycler^®^ Gradient (Eppendorf, Germany) with following conditions: 95 °C for 1 min followed by 30 cycles at 95 °C for 20 s, 56 °C for 15 s, and 72 °C for 40 s with a final extension at 72 °C for 4 min.

PCR products were sequenced at Microsynt Seqlab (Germany). Sequences were analysed with Geneious (v10.2.6) (Biomatters, Auckland, New Zealand). Consensus sequences (approximately 250 bp) were used in a BLAST search against the NCBI nucleotide database [24]. A cut-off value of 99.0% for species and 97.5% for the genus was used in identification.

#### 2.3.3. DNA-Isolation and Amplicon Library Preparation

Total DNA was isolated from fish slurry prepared as described above (Section 2.2), with the difference that homogenisation was done for 120 s at 230 rpm. The 45 mL aliquots of the fish slurry were centrifuged at 200× *g* for 5 min at 4 °C, and supernatants (15 mL) were harvested and further centrifuged at 10,000× *g* for 10 min at 4 °C. Supernatants were discarded and pellets were frozen and stored at −80 °C.

Pellets were suspended in 1.8 mL of 0.9% NaCl, and DNA was extracted with the DNeasy PowerFood Microbial Kit (Qiagen, Hilden, Germany) according to manufacturer instructions. NaCl 0.9% was used as a negative control at the extraction stages. The extracted DNA was quantified with NanoDrop (Thermo Scientific, Wilmington, DE, USA). The eluted DNA was stored at −20 °C.

Due to the high background of matrix (fish) DNA, the bacterial amplicon libraries were generated following a two-step protocol. In the first step, the 16S rRNA gene was amplified with primers BSF 8/20 and BSR 1541/20. Reactions (25 µL) contained 12.5 μL 2 × MyTaqTM Red Mix (Bioline, UK), 0.2 μM of each primer, and 4 μL template. PCR was conducted in a 96-well plate with the following program: pre-PCR heat step for 3 min at 95 °C, 15 cycles at 95 °C for 20 s, 54 °C for 20 s, and 72 °C for 40 s, followed by a final extension at 72 °C for 4 min.

With the first-round PCR products as templates, the second PCR was conducted with primers 341f (barcoded) and 785r [25]. Each reaction was performed in duplicate in a PCR mixture containing 12.5 μL 2× MyTaqTM Red Mix, 0.4 μM of each primer, 4 μL template, and water up to 25 μL. PCR program included initial incubation at 95 °C for 3 min, 35 cycles of 95 °C for 15 s, 50 °C for 30 s, and 72 °C for 15 s, with a final extension at 72 °C for 3 min. PCR grade water was used as negative control throughout the nested PCR.

PCR products were verified with gel electrophoresis. Further library handling and sequencing on the IonTorrent platform were carried out at Bioser (Biocenter Oulu Sequencing Center, Oulu, Finland).

#### 2.3.4. Sequence Processing and Data Analyses

Raw Ion Torrent reads were processed with Mothur version 1.43.0 [26]. Reads were de-multiplexed and quality-filtered: reads with the length shorter than 200 bp and longer than 400 bp, ambiguous bases, homopolymers longer than 8 bp, or Phred score < 25 were removed. Unique sequences were aligned against SILVA reference database release 132 [27] and filtered to remove overhanging sequences at both ends. Reads were pre-clustered to remove sequences that were within a distance of 1 bp per 100 bp of more abundant sequences and hence likely generated due to a sequencing error. UCHIME in *de novo* mode [28] was used to detect chimeric sequences. Classification of denoised reads was done against SILVA (release 132) with a naïve Bayesian classifier [29] with an 80% confidence. Sequences named chloroplast were removed from further analysis. The remaining sequences were clustered into operational taxonomic units (OTUs) with the DGC method [30] with a threshold of 97% similarity. OTUs with only one sequence in all samples were removed, and OTUs were binned to different taxonomic levels. SILVA database taxonomy is employed further in this study.

### 2.4. Statistical Analysis and Visualization

An independent samples *t*-test was conducted with IBM SPSS Statistics 26 (IMB, Armonk, NY, USA) to determine whether levels of aerobic mesophiles, psychrotrophs, H₂S-producers, and enterobacteria differed between whole and gutted samples with a significance level of *p* < 0.05.

Alpha and beta diversities were calculated with Mothur. For assessments of alpha diversities, data were rarefied to 2219 reads per sample. To estimate alpha diversity, the ACE (abundance-based coverage estimator) richness index, the Shannon diversity index, and the inverse Simpson index were calculated. The between-samples diversity (beta diversity) was analysed with the Thetayc calculator. Results were plotted with OriginPro 2020 (OriginLab Corporation, Northampton, MA, USA). A statistically significant difference between sample types was determined by independent samples *t*-test with a significance level of *p* < 0.05.

## 3. Results

### 3.1. Viable Counts

The bacterial counts of whole and gutted herring were analysed at ten different times representing different seasons (Table 2 and Table S1). In the examined batches, the whole and gutted herring had aerobic mesophiles and psychrotrophs in the range of 2.8 log_10_ CFU g^−1^ to 5.3 log_10_ CFU g^−1^. The H₂S-producing bacteria numbers varied from 2.2 log_10_ CFU g^−1^ to 5.6 log_10_ CFU g^−1^. Low levels of enterobacteria and coliforms were detected. Spores, enterococci, and H_2_S-reducing clostridia remained under the detection limit (<1 log_10_ CFU g^−1^ or <2 log_10_ CFU g^−1^). In most of the bacterial groups, gutted herring had lower bacterial counts than whole herring (*p* < 0.05).

### 3.2. Identification of Bacterial Isolates

A total of 813 isolates were analysed with MALDI-TOF MS, 189 of which were picked from the PCA plates incubated at 30 °C, 229 from the PCA plates incubated at 10 °C, 217 from the Chromocult plates, and 178 from the Lyngby plates. Altogether, 671 isolates (82.5%) were identified at genus or species level, and 142 isolates (17.5%) remained unidentified. Selected isolates (*n* = 15) with no genus or species-level identification by MALDI-TOF MS were analysed with 16S rRNA sequencing. Those isolates were identified as members of *Acinetobacter* (*n* = 4), *Flavobacterium* (*n* = 3), *Pseudomonas* (*n* = 3), and five other genera (*Chryseobacterium*, *Comamonas*, *Myroides*, *Shewanella*, and *Wohlfahrtiimonas*) (Table S3).

**Figure 1:**
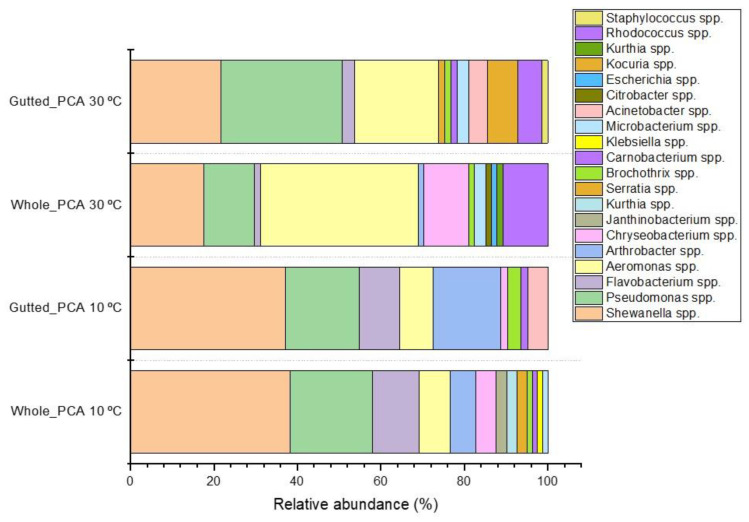
Relative abundance of the dominant bacterial colony types isolated from PCA and identified at genus level with MALDI-TOF or 16S rRNA gene sequencing.

Figure 1 displays bacterial isolates from PCA with genus-level identification. In both sample types, *Shewanella* isolates were abundant on PCA, and the genus became dominant (up to 38.3%) when incubation at 10 °C was applied. Furthermore, the share of *Flavobacterium* spp. and *Arthrobacter* spp. increased when the incubation temperature was lower. The genus *Pseudomonas* was a major part of both the mesophilic and psychrotrophic populations, with the highest abundance in the gutted herring samples incubated at 30 °C, where it represented 29.0% of all the identified colonies. Members of the genus *Aeromonas* were frequently isolated, with a higher incidence in the mesophilic population. In the whole herring, *Aeromonas* dominated at 30 °C, where 37.8% of identified colonies were confirmed as belonging to the genus. *Shewanella* spp. were most frequently isolated from the Lyngby medium, where they were the main H₂S-producers (Table S2).

Altogether, 16 species-level identifications were made with MALDI-TOF MS Biotyper (RUO database) (score value ≥ 2.3): *Aeromonas salmonicida*, *Arthrobacter bergerei*, *Brochothrix thermosphacta*, *Carnobacterium maltaromaticum*, *Chryseobacterium vrystaatense*, *Citrobacter braakii*, *Citrobacter gillenii*, *Escherichia coli*, *Hafnia alvei*, *Lelliottia amnigena*, *Microbacterium maritypicum*, *Morganella morganii*, *Rhodococcus erythropolis*, *Shewanella baltica*, *Staphylococcus hominis*, and *Yersinia enterocolitica*. Moreover, *Acinetobacter albensis* was identified with 16S rRNA gene sequencing.

### 3.3. Culture-Independent Population Analysis

Bacterial population profiling was performed for 70 selected samples. A total of 668,341 quality-filtered sequences were obtained (ranging from 2219 to 28,051 per sample). Sequences were clustered into 6872 OTUs sharing 97% sequence similarity; however, only 1411 OTUs had a minimum of 10 reads per OTU.

**Figure 2:**
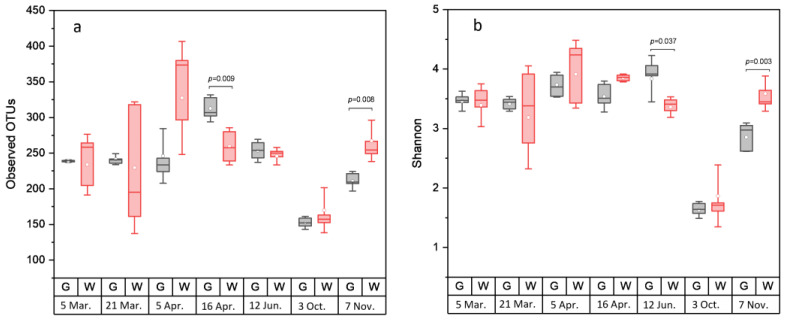
Alpha diversity of bacterial operational taxonomic units (OTUs) in the whole and gutted herring. The number of observed OTUs (**a**) and the Shannon index (**b**), attained from sequence data rarefied to 2219 reads. The boxes represent the interquartile range between the first and third quartiles. The horizontal line inside the box is the median and the white circle the mean, with the standard deviation obtained from the 4 to 6 parallel samples of gutted (G) or whole (W) fish. *p*-value is presented whenever a statistical difference in the mean values between the whole and gutted herring was found.

**Figure 3:**
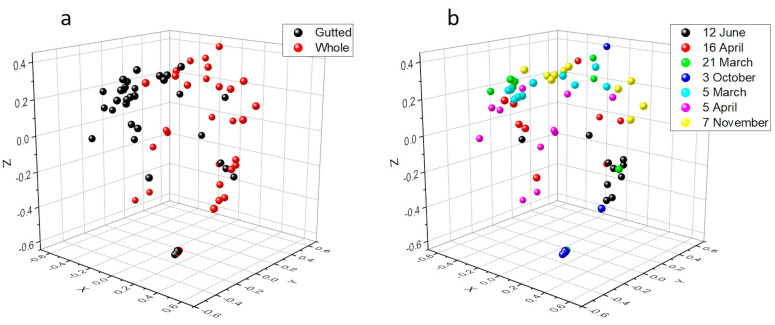
Non-metric multidimensional scaling (NMDS) plot of the OTUs of the whole and gutted herring calculated with the Thetayc distance matrix by the gutting treatment (**a**) and the sampling date (**b**), with 97% similarity threshold. The lowest stress is 0.15 and R^2^ 0.86.

Rarefaction analysis illustrated that the bacterial communities were well-characterized and differences in the observed OTU numbers were largely not caused by variations in sequencing depth (Figure S1). The species richness and the evenness of the within-sample distribution (measured by the Shannon index) were the highest in the 5 April samples and the lowest in the 3 October samples, in the latter of which the dominance of a single genus (*Arcobacter*) was detected (Figure 2 and Figure S2). A difference (*p* < 0.05) between the whole and the gutted herring was observed only for two batches, indicating that species richness and evenness were not consistently influenced by the gutting treatment. The non-metric multidimensional scaling (NDMS) analysis of the between-sample diversity index (Thetayc) showed clustering of the OTUs by sample type (Figure 3) apart from two sampling dates (12 June and 3 October), indicating that the whole and gutted herring had, to a degree, distinct bacterial communities.

**Figure 4:**
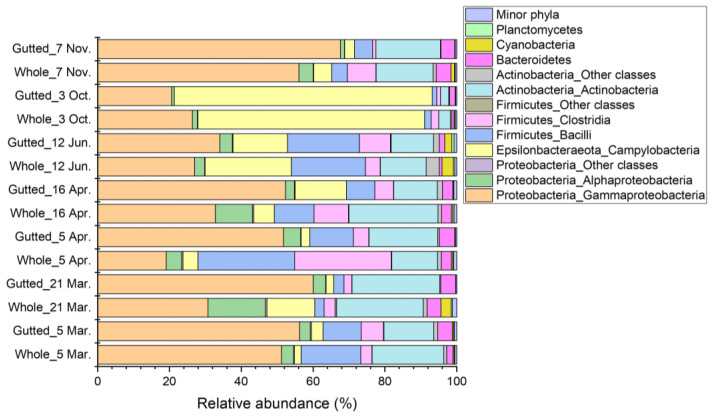
Bacterial population composition of the whole and gutted herring at the class-phylum level as identified with 16S rRNA gene amplicon sequencing. Each batch is an average of four to six whole and gutted fish.

In most of the samples, the majority of the sequences (71.7% to 95.0%) were assigned to the phyla Actinobacteria, Firmicutes, and Proteobacteria (Figure 4). An exception was the 3 October batch, where their share was only 37.5% and 26.1% in the whole and gutted herring, respectively. The dominant phylum observed in this batch was Epsilonbacteraeota, which accounted for up to 72.0% of all sequences. The phylum was present in all other samples as well, yet with lower abundance. Together, these four main phyla made up 92.7% to 98.6% of the bacterial population at all sampling points. The majority of the remaining less than 8% of all sequences were assigned to phyla Bacteroidetes, Cyanobacteria, and Planctomycetes.

**Figure 5:**
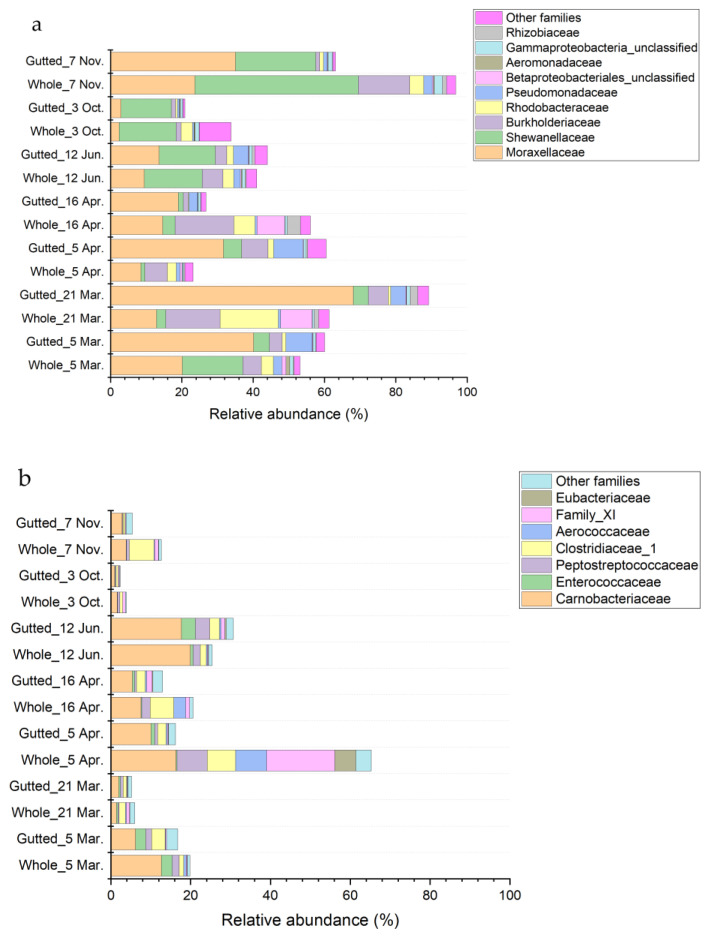

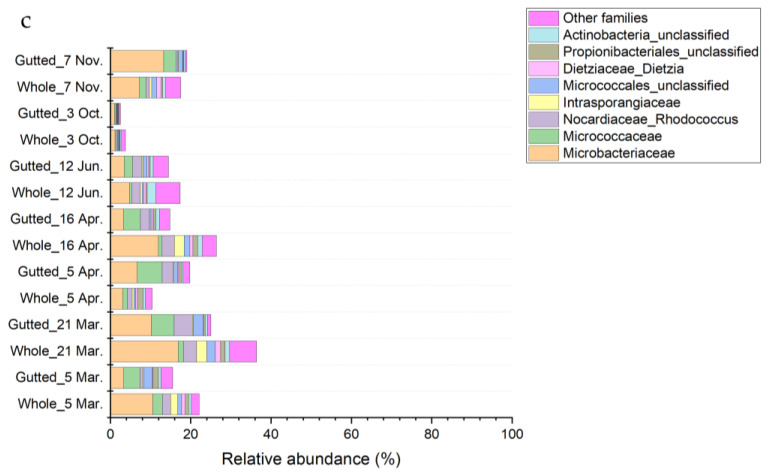
Bacterial population composition of the whole and gutted herring identified with 16S rRNA gene amplicon sequencing. The phyla Proteobacteria (**a**), Firmicutes (**b**), and Actinobacteria (**c**) are presented at the family level. Each batch is an average of four to six whole and gutted fish.

The phylum Proteobacteria was largely represented by the class Gammaproteobacteria except for the whole herring analysed on 21 March and 16 April (Figure 5 and Figure S3). In these batches, other proteobacterial classes, including Alphaproteobacteria, accounted for 13.1% of the total bacterial community at the most. Within Gammaproteobacteria, most of the sequences were assigned to families Moraxellaceae (main genera *Acinetobacter* and *Psychrobacter*) and Shewanellaceae (mainly genus *Shewanella*) and to a lesser extent Burkholderiaceae (genera including, e.g., *Simplicispira* and *Polaromonas*), Rhodobacteriaceae, Pseudomonadaceae (mainly genus *Pseudomonas*), Aeromonadaceae (mainly genus *Aeromonas*), and other minor families. Enterobacteriaceae was among the minor families encompassing only 0.44% of all sequences at the maximum. Most of the sequences assigned to the family were not identified to genus level except for a small minority represented by the genus *Serratia*.

Major classes belonging to the phylum Firmicutes were Clostridia and Bacilli (Figure 5), which together represented 2.3% to 30.7% of the total bacterial community, except for the whole herring on 5 April, where the proportion of the phyla represented up to 65.2% of all the sequences. In that sample, clostridial families Peptostreptococcae (most abundant genera *Proteocatella*, *Peptostreptococcus*, and *Acetoanaerobium*), Family XI (mainly genus *Gottschalkia*), Eubacteriaceae (mainly genus *Acetobacterium*), and Clostridiaceae_1 (most abundant genera *Proteiniclasticum*, Clostridium_sensu_stricto_13, and Clostridium_sensu_stricto_5) accounted for over 30% of the bacterial population. In most samples, the majority of sequences assigned to the phylum Firmicutes belonged to the class Bacilli, in which the family Carnobacteriaceae (mainly genera *Carnobacterium* and *Trichococcus*) was the most represented and covered up to 19.9% of all sequences.

The phylum Actinobacteria was present in all the samples with a 10.4% to 30.8% share except for the 3 October samples, when the phylum was less than 4% of the total population. The phylum was mostly represented by the class Actinobacteria (Figure 5), in which the majority of the sequences belonged to the family Microbacteriaceae (not identified at genus level), Micrococcaceae (mainly genus *Arthrobacter*), and Nocardiaceae (only genus *Rhodococcus*).

Phylum Epsilonbacteraeota was dominant in the 3 October samples and accounted for 13.8% to 23.2% of all sequences in the 21 March whole herring, 16 April gutted herring, and 12 June samples. A total of 98.0% of the sequences assigned to the phylum were represented by the family Arcobacteraceae, in which all the OTUs belonged to the genus *Arcobacter*.

## 4. Discussion

In this study, the microbiological quality of several commercially fished herring batches was analysed. The batches were randomly selected, and they represented different seasons, as the aim of this study was to investigate whether the quality of the whole herring was acceptable for food use throughout the year as such, without further processing. The aerobic mesophilic and psychrotrophic bacteria levels detected in this study were similar to those reported previously for fresh herring aerobically stored in ice [17,18,19,20]. The EU regulation on the microbiological criteria for foodstuff ((EC) No. 1441/2007) lacks guideline values regarding fresh fish. Nevertheless, the Finnish Food and Drink Industries’ Federation has issued microbiological quality recommendations for several food supplies on the last day of use, including fish [31]. The aerobic total counts were within the recommended limit (m < 6.0 log_10_ CFU g^−1^) in all the sampled batches. H₂S-producing bacteria (frequently *Shewanella* spp.) are a well-known group of spoilage micro-organisms in fish [11,32] and were hence examined in this study. The levels of H₂S-producing bacteria slightly exceeded the recommended m-value (m < 5.0 log_10_ CFU g^−1^) in two sample batches, which was not entirely surprising, as they were examined only a day before the expiry date.

The gutted herring was of higher microbial quality than the whole herring, as indicated by most of the examined bacterial groups in the majority of the sampling points (*p* < 0.05). Nevertheless, the microbiological quality of the small-sized whole herring was also at an acceptable level. Similar results have been reported previously for whole and gutted sardine and sea bass [33,34]. However, some studies have found increased bacterial counts in gutted fish as opposed to whole fish, presumably due to increased handling and fish flesh exposure to the gutting machines [10,35]. In this study, microbial numbers could not be attributed to seasonal variation, as diverse levels were observed within the same season. This is consistent with the findings of a previous paper on the chemical and microbiological quality of Mediterranean horse mackerel, where the authors proposed that the fishing environment, method of catching, and onboard handling affected the quality more than the season [36]. In the present study, the method of catching was consistent, but other factors, such as the fishing area or possible fluctuation in the water quality, may have impacted the microbiological quality.

This is, to the authors’ knowledge, the first study to examine the microbiota of Baltic herring to this extent by combining culture-based methods and high-throughput amplicon sequencing. With both approaches, the bacterial class Gammaproteobacteria dominated the microbiota of the whole and the gutted herring. The dominance of this taxon in the microbiota of various fish species has been widely described earlier [7,37,38,39,40]. Within the bacterial class, the genera *Shewanella*, *Pseudomonas*, and *Aeromonas* were the most frequent isolates among the mesophilic and psychrotrophic populations on PCA. The bacterial population analyses based on the 16S rRNA gene amplicon sequencing revealed that among the most abundant genera were *Shewanella* and *Psychrobacter*, with up to 47.1% and 34.8% of all the sequences assigned to the genera at some sampling points, respectively. *Psychrobacter* has been reported to dominate the microflora of the Atlantic mackerel and gilt-head sea bream examined with culture-independent methods [7,41]. In the present study, *Psychrobacter* was never isolated yet turned out to be among the most abundant genera detected by 16S rRNA gene amplicon sequencing. The limitations in the MALDI-TOF reference database could account for the low identification rate of *Psychrobacter*, as has been earlier noted by other authors regarding poultry isolates [14]. Another dissenting result between culturing and 16S rRNA gene amplicon sequencing was the low relative abundance of the genera *Pseudomonas* and *Aeromonas*. Although the genera were detected in all the samples, they remained in the minority, with a maximum of 7.6% and 1.1% of the sequences assigned to the genera, respectively. The high abundance of *Pseudomonas* and *Aeromonas* isolates could be explained by the bias introduced by culturing. These organisms are favoured by the culturing conditions and grow faster than those with lower growth rates, increasing the likelihood of being isolated from the media [16]. Within the class Gammaproteobacteria, the level of Enterobacteriaceae, an important hygiene indicator in foods, was low in all the sampled fish batches with culture-dependent methods. Additionally, with 16S rRNA gene amplicon sequencing, the bacterial family was a minority, encompassing only 0.44% of all sequences at the maximum.

Overall, the relative abundance of the phylum Proteobacteria was higher in the gutted herring compared to the whole herring, as a higher proportion of sequences assigned to the family Moraxellaceae were detected in the samples. Additionally, the phylum Firmicutes was represented with higher relative abundance in the gutted herring samples, except for the fish analysed on 12 June. Nevertheless, major differences between the two sample types were not observed. The 3 October sample batch was surprisingly different from the other batches because of the high relative abundance of the phylum Epsilonbacteraeota. This group refers to a subclass of the phylum Proteobacteria, which currently is named Epsilonproteobacteria [42] but has been proposed to be elevated as a new phylum [43]. The known members of the bacterial group are the pathogenic genera *Campylobacter* and *Helicobacter*, as well as an emerging opportunistic food-borne pathogen, *Arcobacter* [44,45]. The genus *Arcobacter* is highly abundant in wastewater and has been isolated from coastal waters, seafood (including fish and shellfish), and foodstuff of an animal origin [46,47]. *Arcobacter* was detected in all the herring samples in the present study, particularly in the 3 October batch, where it was the major group. Since there was only one sample batch in which this genus dominated the whole bacterial community, and no data on water quality are available, it is difficult to draw a certain conclusion. Therefore, more studies are needed on the association between the environmental parameters, water quality, and fish microbial load.

To conclude, our study revealed the microbial quality of the whole herring batches fished between September to June was similar to the gutted herring. Results obtained from combining the culture-dependent and independent approaches show that the microbiota of the herring was dominated by the bacterial class Gammaproteobacteria, yet with noticeable method-specific differences in the species abundance. The whole herring showed acceptable microbiological quality throughout the sampling period, which supports the use of underutilized fish in the food industry. However, whole fish is highly perishable, which poses challenges to its further processing. Therefore, the fishing industry would benefit from the development of technologies (e.g., on-board cooling and on-site quality detection systems) to ensure the high quality and usability of the small-size low-value fish in food production.

## Figures and Tables

**Table 1:** Media used for direct enumeration of bacteria.

Bacterial Group	Culture Medium	Incubation Conditions
Aerobic mesophilic bacteria	Plate Count Agar (PCA) (BD Difco, Franklin Lakes, NJ, USA)	Aerobic, 30 °C, 3 days
Aerobic psychrotrophic bacteria	Plate Count Agar (PCA) (BD Difco, Franklin Lakes, NJ, USA)	Aerobic, 10 °C, 7 days
Spores ^1^	Plate Count Agar (PCA) (BD Difco, Franklin Lakes, NJ, USA)	Aerobic, 30 °C, 3 days
*Bacillus cereus*	Mannitol Egg Yolk Polymyxin agar (Oxoid, Hampshire, UK)	Aerobic, 37 °C, 24 h
Hydrogen sulphide-producing bacteria	Lyngby iron agar ^2^ (Oxoid, Hampshire, UK)	Aerobic, 25 °C, 2 days
Anaerobic sulphide-reducing clostridia	Sulphite iron agar (BioLab, Tampere, Finland)	Anaerobic ^3^, 37 °C, 2 days
Lactic acid bacteria (LAB)	De Man Rogosa Sharpe agar (MRS) (Oxoid, Hampshire, UK)	Anaerobic ^3^, 25 °C, 5 days
Enterococci	mEnterococcus agar (BD Difco, Franklin Lakes, NJ, USA)	Aerobic, 37 °C, 2 days
*Pseudomonas* spp. and *Aeromonas* spp.	GSP selective agar (Merck, Darmstadt, Germany)	Aerobic, 28 °C, 3 days
Enterobacteria ^4^	Violet Red Bile Glucose Agar (LabM, Lancashire, UK)	Aerobic, 37 °C, 24 h
Coliforms	Chromocult coliform agar (Merck, Darmstadt, Germany)	Aerobic, 37 °C, 24 h

^1^ Heat treatment 80 °C, 10 min. ^2^ With 4% L-cysteine supplementation. ^3^ Anaerobic conditions (N₂ 85%, CO₂ 5%, and H₂ 10%) were generated with Anoxomat (Mart Microbiology BV, the Netherlands). ^4^ With the pour plate technique.

**Table 2:** Aerobic mesophilic bacteria, aerobic psychrotrophic bacteria, H₂S-producing bacteria, and enterobacteria counts in whole and gutted herring fished at different seasons are presented as log_10_ CFU g^−1^. The remaining shelf life of the fish batches is shown in days.

Microbiological Sampling Date	Remaining Shelf Life		Aerobic Mesophiles		Aerobic Psychrotrophs		H_2_S-Producers		Enterobacteria	
	Whole	Gutted	Whole	Gutted	Whole	Gutted	Whole	Gutted	Whole	Gutted
6 March	1	3	4.9 ± 0.3	3.8 ± 0.1	5.3 ± 0.2 ^a^	4.4 ± 0.7 ^a^	5.5 ± 0.4	4.0 ± 0.1	<1 ^b^	1.0 ± 1.0 ^b^
21 March	2	4	3.5 ± 0.1 ^c^	3.5 ± 0.1 ^c^	4.0 ± 0.2 ^d^	3.9 ± 0.1 ^d^	2.4 ± 0.1 ^e^	2.2 ± 0.4 ^e^	2.2 ± 0.9 ^f^	1.0 ± 0.0 ^f^
5 April	1	4	4.1 ± 0.4	3.3 ± 0.1	4.6 ± 0.2	3.7 ± 0.2	3.4 ± 0.2 ^g^	3.4 ± 0.4 ^g^	<1 ^h^	<1 ^h^
16 April	Nd ^1^	Nd ^1^	3.3 ± 0.2	2.8 ± 0.2	4.3 ± 0.3	3.3 ± 0.2	4.0 ± 0.5	3.4 ± 0.2	<1	1.7 ± 0.1
14 May	2	5	3.3 ± 1.8 ^i^	3.0 ± 0.1 ^i^	4.3 ± 0.1	3.1 ± 0.3	4.1 ± 0.1	2.9 ± 0.3	1.1 ± 0.7	<1
12 June	1	4	4.6 ± 0.4	3.5 ± 0.2	4.9 ± 0.3	3.9 ± 0.2	5.0 ± 0.3	4.0 ± 0.3	1.9 ± 0.6 ^j^	1.7 ± 0.1 ^j^
3 October	3	Na ^2^	4.2 ± 0.1	3.8 ± 0.1	4.4 ± 0.2	4.0 ± 0.2	4.6 ± 0.4	3.8 ± 0.2	2.0 ± 0.4 ^k^	1.0 ± 1.0 ^k^
7 November	1	3	4.3 ± 0.1 ^l^	4.0 ± 0.4 ^l^	4.5 ± 0.1 ^m^	4.5 ± 0.3 ^m^	5.5 ± 0.2 ^o^	5.6 ± 0.2 ^o^	<1 ^p^	<1 ^p^
29 May	2	5	4.0 ± 0.2	3.2 ± 0.2	4.2 ± 0.1	3.5 ± 0.2	4.0 ± 0.1	3.5 ± 0.1	<1 ^q^	<1 ^q^
20 November	3	Na ^2^	4.6 ± 0.2	4.1 ± 0.1	4.7 ± 0.3 ^r^	4.5 ± 0.2 ^r^	4.6 ± 0.5 ^s^	4.6 ± 0.2 ^s^	1.1 ± 0.7 ^t^	1.1 ± 0.2 ^t^

^1^ Nd = No data. ^2^ Na = Not applicable. The batch was gutted manually in the laboratory. ^a–m,o–t^ No statistically significant difference in mean values between whole and gutted herring within the same bacterial group and sampling point (*p* > 0.05). Mean values not sharing letters differ significantly.

## Data Availability

Data is contained within the article or supplementary material.

## Supplementary Materials

The following are available online at https://www.mdpi.com/article/10.3390/foods11040492/s1, Table S1: Coliform, spore, *Bacillus cereus*, lactic acid bacteria, enterococci, *Pseudomonas* spp., *Aeromonas* spp., and anaerobic sulphide-reducing clostridia counts in whole and gutted herring batches presented as log10 CFU g−1, Table S2. Number (n.) and percentage (%) of bacterial isolates from PCA incubated at 10 °C (a) or 30 °C (b), Chromocult agar (c), and Lyngby (d) agar identified at the genus level with MALDI-TOF MS and 16S rRNA gene sequencing, Table S3: Bacterial isolates identified with 16S rRNA gene sequencing, Figure S1: Rarefaction curves of the sequence data normalized to the equal number of reads per sample at a 97% similarity threshold, Figure S2. Alpha diversity of bacterial OTUs in the whole and gutted herring from seven different batches. Inverse Simpson index (a) Shannon index (b), the number of observed OTUs (c), and ACE (d) attained from sequence data rarefied to 2219 reads, Figure S3: Bacterial population composition of the whole and gutted herring at the class level as identified with 16S rRNA amplicon sequencing.
