# Supplementary material for: Characterization of Microbiological Quality of Whole and Gutted Baltic Herring
Source: Foods. 2022 Feb 9;11(4):492. doi: 10.3390/foods11040492 (PMC8871270; doi:10.3390/foods11040492)
Supplement: Supplementary file 1 [file foods-11-00492-s001.zip › foods-1564917-supplementary.pdf]

Supplementary

Table S1. Coliform, spore, *Bacillus cereus*, lactic acid bacteria, enterococci, *Pseudomonas* spp., *Aeromonas* spp., and anaerobic sulphide reducing clostridia counts in whole and gutted herring batches presented as log_{10} CFU g^{-1}.

**Microbiological sampling date**	**Coliforms**		**Spores**		***Bacillus cereus***		**LAB**	
	**Whole**	**Gutted**	**Whole**	**Gutted**	**Whole**	**Gutted**	**Whole**	**Gutted**
Mar. 6	<1	<1	-	-	-	-	-	-
Mar. 21	<1	1.0±0.0	-	-	-	-	-	-
Apr. 5	<2	<2	<1	<1	-	-	-	-
Apr. 16	<1	<1	<2	<2	-	-	-	-
May 14	2.2±0.1	<1	<1	<1	-	-	-	-
Jun. 12	1.5±1.0	<1	<1	<1	-	-	-	-
Oct. 3	<1	<1	<1	<1	-	-	2.5±0.2	2.0±0.2
Nov. 7	<1	<1	<1	<1	-	-	1.9±0.7	1.7±0.6
May 29	<1	<1	<1	<1	<1	<1	2.5±0.3	2.1±0.6
Nov. 20	<1	<1	<1	<1	-	-	-	-

**Sampling point**	**Enterococci**		***Pseudomonas***		***Aeromonas***		**Anaerobic clostridia**	
	**Whole**	**Gutted**	**Whole**	**Gutted**	**Whole**	**Gutted**	**Whole**	**Gutted**
Mar. 6	-	-	-	-	-	-	-	-
Mar. 21	-	-	-	-	-	-	-	-
Apr. 5	-	-	-	-	-	-	-	-
Apr. 16	-	-	-	-	-	-	-	-
May 14	-	-	-	-	-	-	-	-
Jun. 12	-	-	-	-	-	-	-	-
Oct. 3	<1	<1	-	-	-	-	<1	<1
Nov. 7	<1	<1	-	-	-	-	<1	<1
May 29	<1	<1	-	-	-	-	-	-
Nov. 20	-	-	3.6±0.3	3.4±0.3	3.7±0.8	3.1±0.2	<1	<1

*May 14th coliforms were not estimated based on the percentage of colonies confirmed as coliforms by MALDI-TOF MS as in other sampling dates.

Table S2. Number (n.) and percentage (%) of bacterial isolates from PCA incubated at 10 °C (a) or 30 °C (b), Chromocult agar (c), and Lyngby (d) agar identified at the genus level with MALDI-TOF MS and 16S rRNA gene sequencing.

a

PCA 10 °C

Whole

Bacterial identification	n.	%
Aeromonas spp.	6	7,4
Arthrobacter spp.	5	6,2
Brochothrix spp.	1	1,2
Carnobacterium spp.	1	1,2
Chryseobacterium spp.	4	4,9
Flavobacterium spp.	9	11,1
Janthinobacterium spp.	2	2,5
Klebsiella spp.	1	1,2
Kurthia spp.	2	2,5
Microbacterium spp.	1	1,2
Pseudomonas spp.	16	19,8
Serratia spp.	2	2,5
Shewanella spp.	31	38,3
	81	100

Gutted

Bacterial identification	n.	%
Acinetobacter spp.	3	4,8
Aeromonas spp.	6	8,1
Arthrobacter spp.	13	16,1
Brochothrix spp.	2	3,2
Carnobacterium spp.	1	1,6
Chryseobacterium spp.	1	1,6
Flavobacterium spp.	11	9,7
Pseudomonas spp.	18	17,7
Shewanella spp.	23	37,1
	78	100

b

PCA 30 °C

Whole

Bacterial identification	n.	%
Aeromonas spp.	28	37,8
Arthrobacter spp.	1	1,4
Brochothrix spp.	1	1,4
Chryseobacterium spp.	8	10,8
Citrobacter spp.	1	1,4
Flavobacterium spp.	1	1,4
Kurthia spp.	1	1,4
Microbacterium spp.	2	2,7
Pseudomonas spp.	9	12,2
Rhodococcus spp.	8	10,8
Shewanella spp.	13	17,6
Escherichia spp.	1	1,4
	74	100

Gutted

Bacterial identification	n.	%
Acinetobacter spp.	3	4,3
Aeromonas spp.	14	20,3
Brochothrix spp.	1	1,4
Carnobacterium spp.	1	1,4
Flavobacterium spp.	2	2,9
Kocuria spp.	5	7,2
Microbacterium spp.	2	2,9
Pseudomonas spp.	20	29,0
Rhodococcus spp.	4	5,8
Serratia spp.	1	1,4
Shewanella spp.	15	21,7
Staphylococcus spp.	1	1,4
	69	100

c

Chromocult

Whole

Bacterial identification	n.	%
Aeromonas spp.	89	74,2
Buttiauxella spp.	2	1,7
Citrobacter spp.	9	7,5
Leclercia spp.	1	0,8
Lelliottia spp.	1	0,8
Morganella spp.	1	0,8
Pantoea spp.	4	3,3
Proteus spp.	2	1,7
Staphylococcus spp.	5	4,2
Yersinia spp.	1	0,8
Escherichia spp.	5	4,2
	120	100

Gutted

Bacterial identification	n.	%
Aeromonas spp.	86	88,7
Citrobacter spp.	1	1,0
Hafnia spp.	1	1,0
Serratia spp.	3	3,1
Yersinia spp.	1	1,0
Staphylococcus spp.	5	5,2
	97	100

d

Lyngby [1]

Whole

Bacterial identification	n.	%
Aeromonas spp.	6	7,1
Janthinobacterium spp.	1	1,2
Pseudomonas spp.	6	7,1
Shewanella spp.	70	83,3
Stenotrophomonas spp.	1	1,2
	84	100

Gutted

Bacterial identification	n.	%
Acinetobacter spp.	2	2,9
Aeromonas spp.	3	4,4
Arthrobacter spp.	2	2,9
Brochothrix spp.	1	1,5
Chryseobacterium spp.	1	1,5
Kurthia spp.	1	1,5
Proteus spp.	1	1,5
Pseudomonas spp.	3	4,4
Shewanella spp.	53	77,9
Stenotrophomonas spp.	1	1,5
	68	100

[1] From Lyngby, not only typical H_2S-producers (black colonies) but also other bacterial colonies were picked for MALDI-TOF identification. Of all identified colonies, 65.8% were H_2S-producers majority of which were *Shewanella* isolates. Additionally, a few isolates identified as *Aeromonas* spp. produced black colonies on the medium.

Table S3. Bacterial isolates identified with 16S rRNA gene sequencing.

Query cover	Per.ident.	Accession	Description
100 %	98.33%	NR_025267.1	*Shewanella baltica* strain 63 16S ribosomal RNA, partial sequence
99 %	96.99%	NR_145641.1	*Acinetobacter albensis* strain ANC 4874 16S ribosomal RNA, partial sequence
99 %	99.90%	NR_145641.1	*Acinetobacter albensis* strain ANC 4874 16S ribosomal RNA, partial sequence
99 %	99.90%	NR_145641.1	*Acinetobacter albensis* strain ANC 4874 16S ribosomal RNA, partial sequence
100 %	98.96%	NR_116388.1	*Pseudomonas caeni* strain HY-14 16S ribosomal RNA, partial sequence
100 %	97.59%	NR_117475.1	*Myroides phaeus* strain MY15 16S ribosomal RNA, partial sequence
100 %	99.60%	NR_116388.1	*Pseudomonas caeni* strain HY-14 16S ribosomal RNA, partial sequence
99 %	97.43%	NR_133893.1	*Wohlfahrtiimonas larvae* strain KBL006 16S ribosomal RNA, partial sequence
98 %	98.13%	NR_025080.1	*Comamonas denitrificans* strain 123 16S ribosomal RNA, partial sequence
100 %	99.91%	KT825519.1	*Pseudomonas japonica* NBRC 103040 = DSM 22348 16S ribosomal RNA gene, partial sequence
99 %	97.68%	NR_112814.1	*Flavobacterium frigidarium* strain NBRC 102676 16S ribosomal RNA, partial sequence
93 %	98.51%	MH100900.1	*Flavobacterium luteum* strain P3160 16S ribosomal RNA gene, partial sequence
91 %	98.72%	NR_118474.1	*Flavobacterium hercynium* strain DSM 18292 16S ribosomal RNA, partial sequence
99 %	99.81%	NR_145641.1	*Acinetobacter albensis* strain ANC 4874 16S ribosomal RNA, partial sequence
99 %	99.56%	NR_042370.1	*Chryseobacterium vrystaatense* strain R-23566 16S ribosomal RNA, partial sequence

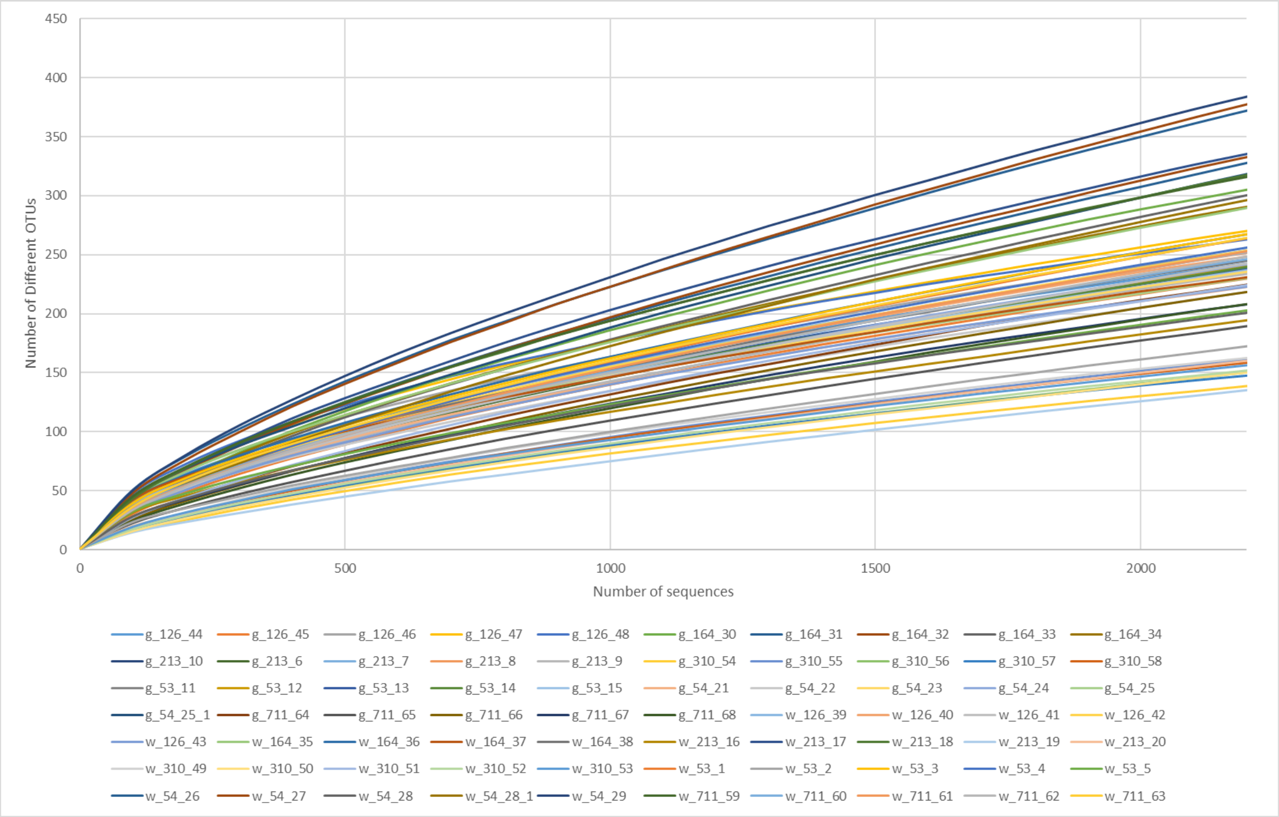

Figure S1. Rarefaction curves of the sequence data normalized to the equal number of reads per sample at a 97% similarity threshold. The letter g in the sample name refers to gutted herring and w to the whole herring. The number after the letter indicates the microbiological sampling date, e.g. 126 means June 12th.

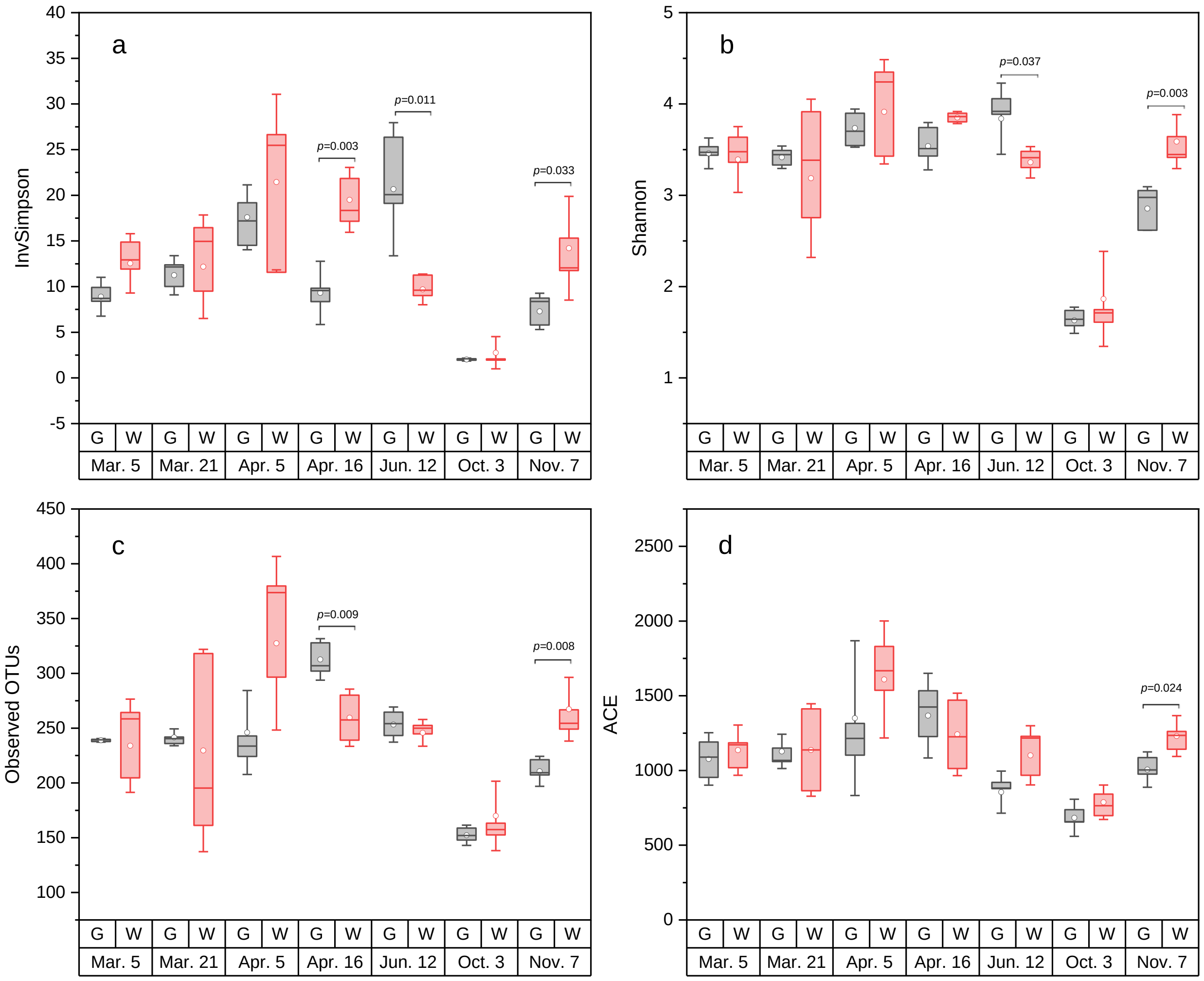

Figure S2. Alpha diversity of bacterial OTUs in the whole and gutted herring from seven different batches. Inverse Simpson index (a) Shannon index (b), the number of observed OTUs (c), and ACE (d) attained from sequence data rarefied to 2219 reads. The boxes represent the interquartile range between the first and third quartiles. The horizontal line inside the box is the median and the white circle the mean with the standard deviation obtained from the 4 to 6 parallel samples. P-value is presented whenever a statistical difference in the mean values between the whole and gutted herring was found.

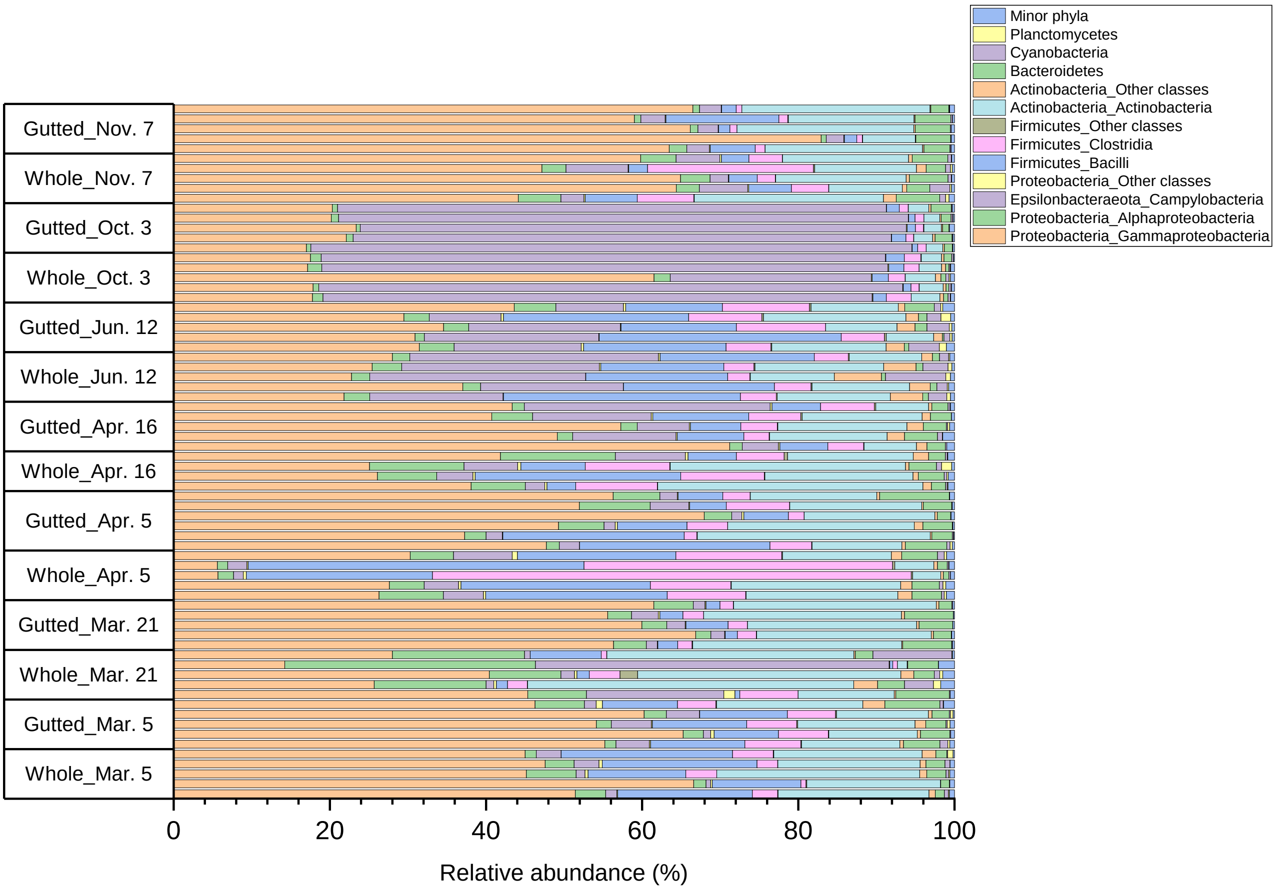

Figure S3. Bacterial population composition of the whole and gutted herring at the class level as identified with 16S rRNA amplicon sequencing. Each batch is represented by four to six whole and gutted fish.
